# Pacing-facilitated Short–long–short Sequences Leading to Ventricular Tachyarrhythmias: A Brief Report

**DOI:** 10.19102/icrm.2024.15093

**Published:** 2024-09-15

**Authors:** Hafez Golzarian, Mohammad Shaikh, Fayaz A. Hakim

**Affiliations:** 1 Internal Medicine Residency, Mercy Health St. Rita’s Medical Center, Lima, OH, USA; 2 Heart Rhythm Services, Division of Cardiovascular Diseases, St. Rita’s Medical Center, Lima, OH, USA

**Keywords:** Implantable cardioverter-defibrillator, premature ventricular complex, short–long–short sequence, ventricular fibrillation, ventricular tachycardia

## Abstract

Pacing-induced recurrent short–long–short sequences constitute an important yet overlooked mechanism for triggering ventricular tachyarrhythmias in patients with cardiovascular implantable electric devices. A careful and thorough retrospective analysis of patients’ electrograms allows for a timely diagnosis with appropriate management.

## Case presentation

**Figure 1:**
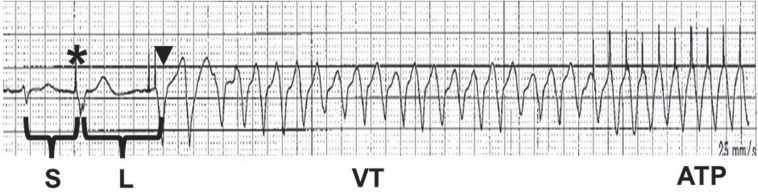
Telemetry recording shows an early coupled premature ventricular complex (asterisk), a rescue pacing event (arrowhead), and onset of ventricular tachycardia. Anti-tachycardia pacing can be observed toward the end of the tracing.

**Figure 2:**
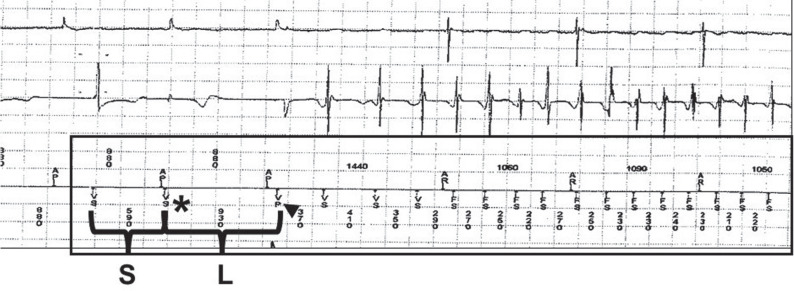
Electrogram showing a premature ventricular complex (590-ms interval; asterisk) that occurs almost simultaneously with atrial pacing (980-ms interval) and leads to a short (S) cycle length. The premature ventricular complex is marked as a sensed event, as it falls in crosstalk windows, and is recognized as a true ventricular event. The next atrial pacing event occurs at an appropriate time (980-ms interval), and a rescue ventricular-paced event (arrowhead) with a narrow atrioventricular delay is delivered, leading to a long (L) cycle length that initiates fast ventricular tachycardia, which is sensed as ventricular fibrillation (F).

A 79-year-old man with ischemic cardiomyopathy (left ventricular ejection fraction, 30%) and a dual-chamber implantable cardioverter-defibrillator (ICD) implanted 5 years ago for primary prevention of sudden cardiac death presented to the emergency department with recurrent palpitations and ICD shocks. Laboratory workup results, including renal function, serum electrolytes, and high-sensitive troponin, were all within normal limits. Electrocardiography revealed isolated premature ventricular complexes (PVCs). A transthoracic echocardiogram was unchanged from the previous study. Coronary angiography findings were also unremarkable, except for a patent stent in the left anterior descending artery. He was started on oral amiodarone and discharged home with instructions to follow up at the outpatient electrophysiology clinic. Two weeks later, he presented again with recurrent ICD shocks and was readmitted for evaluation. The following day, a wide complex tachycardia was captured on telemetry **(Figure 1)**; the corresponding ICD-stored electrogram is shown in **Figure 2**.

The ICD (Virtuoso; Medtronic Inc., Minneapolis, MN, USA) was programmed as “managed ventricular pacing” (MVP) (AAIR <=> DDD/R at 60–100 bpm; upper tracking rate, 120 bpm; pace/sensed atrioventricular [AV] delay, 180/150 ms; and mode switch for atrial rates, >170 bpm). The ventricular-arrhythmia detection zones were programmed as follows: (1) the ventricular tachycardia (VT) detection zone was 150–188 bpm (for 20 beats monitored only) and (2) the ventricular fibrillation (VF) detection zone was >188 bpm. Therapies were programmed as follows: anti-tachycardia pacing (ATP) during charging ×1, then 24-J shock ×1, followed by 35-J shock ×1.

Device interrogation demonstrated several episodes of VT (VF zone) successfully treated with 24-J shocks. All VT/VF episodes were triggered by short–long–short (S–L–S) cycle-length sequences initiated by ventricular pacing triggered by the MVP algorithm following a post-PVC pause (please refer to the caption of **Figure 2** for further explanation). The underlying rhythm of the patient was sinus (resting rate, 74–80 bpm) with a normal AV conduction time (200 ms). The VT/VF episodes were suggestive of pacing-facilitated events. As the patient had normal sinus and AV conduction, the device was programmed as DDD 40–110 pulses/min, and the VF detection rate was increased to 207 bpm. Amiodarone was discontinued, and the dose of metoprolol succinate was maximized. He was discharged with a Holter monitor, which showed a PVC burden of only 5.6%. At 12-month follow-up, he remained free from VT/VF.

Consent was obtained from the subject involved in this study.

## Discussion

Long-term right ventricular pacing has been associated with increased heart failure admission and atrial fibrillation.^1^ Different algorithms have been devised by device companies to minimize ventricular pacing in patients with cardiovascular implantable electric devices (CIEDs). MVP is an algorithm in the Medtronic pacing system that primarily provides atrial-based pacing by operating in an AAI(R) mode while providing dual-chamber (DDDR) pacing during transient or permanent loss of AV conduction.^2^ When the device does not sense an intrinsic R-wave at the end of programmed maximum AV delay, it delivers safety pacing with a short AV delay (80 ms), while, if AV conduction is lost for 2/4 consecutive atrial events, it switches to the DDD(R) mode. It then periodically searches for AV conduction by prolonging the AV delay, and, if intrinsic AV conduction is detected, it switches over to the AAA(R) mode.

An abrupt change in the heart rate, as seen during the S–L–S sequences, is known to initiate VT/VF by promoting a re-entry.^3^ This phenomenon has been reported in patients with a pacemaker programmed as DDD(R), VVI(R), or MVP. The S–L–S sequences in patients with CIEDs can be pacing-permitted (S–L–S sequences without ventricular pacing) or pacing-facilitated (S–L–S sequences actively facilitated by ventricular pacing including the terminal beat after the pause).^1^ Pacing-facilitated S–L–S VT/VF has been reported to occur in 2.6% (MVP), 3.3% (VVI/R), and 5.2% (DDD/R) of patients with VT/VF episodes, respectively.^3^

A careful analysis of the device-stored electrogram, especially at the onset of ventricular arrhythmia, is crucial for proper diagnosis and management. An electrocardiogram or even telemetry tracing obtained during an episode of VT/VF may provide useful clues for diagnosis, as illustrated by this case. Pacing-facilitated S–L–S sequence-triggered ventricular arrhythmias may be difficult to manage and may require deactivation of pacing, which may not be desirable in some patients. Switching pacing modes may not necessarily eliminate the problem. Increasing the lower rate in patients who require pacing may be helpful in preventing S–L–S sequences but at the risk of worsening heart failure. Conduction system pacing would be a desirable option in such patients. As our patient had a normal sinus rate and intact AV nodal conduction, switching the mode to DDD and decreasing the lower rate were effective. Turning on rate smoothing in addition to achieving PVC suppression by anti-arrhythmic agents or even PVC ablation should be considered in non–pacing-related PVC-induced S–L–S sequences.^4^

## Conclusion

Pacing-related S–L–S sequence is an important mechanism for triggering VT and VF. A careful analysis of electrograms allows correct diagnosis and appropriate management.

## References

[1] Sweeney MO, Hellkamp AS (2006). Heart failure during cardiac pacing. Circulation.

[2] Gillis AM, Pürerfellner H, Israel CW (2006). Reducing unnecessary right ventricular pacing with the managed ventricular pacing mode in patients with sinus node disease and AV block. Pacing Clin Electrophysiol.

[3] Sweeney MO, Ruetz LL, Belk P, Mullen TJ, Johnson LW, Sheldon T (2007). Bradycardia pacing-induced short-long-short sequences at the onset of ventricular tachyarrhythmias: a possible mechanism of proarrhythmia?. J Am Coll Cardiol.

[4] Friedman PA, Jalal S, Kaufman S (2006). Effects of a rate smoothing algorithm for prevention of ventricular arrhythmias: results of the Ventricular Arrhythmia Suppression Trial (VAST). Heart Rhythm.

